# Modulation of Neutrophil Apoptosis by Antimicrobial Peptides

**DOI:** 10.5402/2012/345791

**Published:** 2012-03-27

**Authors:** Isao Nagaoka, Kaori Suzuki, François Niyonsaba, Hiroshi Tamura, Michimasa Hirata

**Affiliations:** ^1^Department of Host Defense and Biochemical Research, Juntendo University, Graduate School of Medicine, 2-1-1 Hongo, Bunkyo-ku, Tokyo 113-8421, Japan; ^2^Atopy (Allergy) Research Center, Juntendo University, Graduate School of Medicine, Tokyo 113-8421, Japan; ^3^Seikagaku Biobusiness Corporation, Tokyo 104-0033, Japan; ^4^Institute of Ohtaka Enzyme Co., Hokkaido 047-0156, Japan

## Abstract

Peptide antibiotics possess the potent antimicrobial activities against invading microorganisms and contribute to the innate host defense. Human antimicrobial peptides, *α*-defensins (human neutrophil peptides, HNPs), human *β*-defensins (hBDs), and cathelicidin (LL-37) not only exhibit potent bactericidal activities against Gram-negative and Gram-positive bacteria, but also function as immunomodulatory molecules by inducing cytokine and chemokine production, and inflammatory and immune cell activation. Neutrophil is a critical effector cell in host defense against microbial infection, and its lifespan is regulated by various pathogen- and host-derived substances. Here, we provided the evidence that HNP-1, hBD-3, and LL-37 cannot only destroy bacteria but also potently modulate (suppress) neutrophil apoptosis, accompanied with the phosphorylation of ERK-1/-2, the downregulation of tBid (an proapoptotic protein) and upregulation of Bcl-x_L_ (an antiapoptotic protein), and the inhibition of mitochondrial membrane potential change and caspase 3 activity, possibly via the actions on the distinct receptors, the P2Y_6_ nucleotide receptor, the chemokine receptor CCR6, and the low-affinity formyl-peptide receptor FPRL1/the nucleotide receptor P2X_7_, respectively. Suppression of neutrophil apoptosis results in the prolongation of their lifespan and may be advantageous for the host defense against bacterial invasion.

## 1. Introduction

Neutrophils play an important role as an effector of inflammation, tissue injury, and host defense against microbial infection [1]. The lifetime of neutrophils, terminally differentiated blood cells, is relatively short, and they constitutively undergo apoptosis [1]. Apoptotic neutrophils are phagocytosed by macrophages without release of proinflammatory mediators, leading to the limitation of tissue injury and resolution of inflammatory process [2–4]. In this context, it is interesting to note that spontaneous apoptosis of neutrophils is inhibited in patients with sepsis, systemic inflammatory syndrome (SIRS), and acute respiratory distress syndrome (ARDS) by the action of various pathogen- and host-derived substances, such as bacterial products (i.e., Gram-negative lipopolysaccharide; LPS), cytokines, and chemokines (i.e., IL-1*β* and IL-8) [3, 5–8]. The suppressed neutrophil apoptosis results in the prolongation of their life span and causes the uncontrolled release of cytotoxic metabolites and proinflammatory substances (i.e., reactive oxygen species and proteases), which leads to the amplification of systemic inflammation, tissue injury, and organ failure observed in those disorders [9, 10]. In contrast, neutrophil apoptosis can be accelerated by Fas ligand, reactive oxygen species, immune complexes, and bacterial toxins (such as *Pseudomonas aeruginosa *exotoxin, pyocyanin) produced at the sites of inflammation and infection [11–14]. Inappropriate induction of neutrophil apoptosis is likely to deplete neutrophil numbers and functions, thereby impairing host defense and favoring bacterial invasion and persistence.

Mammalian cells express a number of antimicrobial peptides (host defense peptides) that function as effector components in the innate host defense system [15]. They are found in blood, secretions, epithelial tissues as well as in neutrophil granules and exhibit potent antimicrobial activities against a broad spectrum of invading microorganisms, including both Gram-positive and Gram-negative bacteria, fungi, and viruses [16–20]. Among these peptides, defensins and cathelicidin are considered as the two major classes of antimicrobial peptides in humans [16–20]. Defensins are characterized by the six-cysteine residues forming three intramolecular disulfide bridges and are divided into *α*- and *β*-defensins based on the distribution of cysteines and the linkages of disulfide bonding [16, 17, 19, 20]. Human *α*-defensins are found in neutrophils and the Paneth cells of the small intestine, whereas human *β*-defensins (hBDs) are mainly expressed by epithelial tissues. In contrast, cathelicidins are a family of antimicrobial peptides, characterized by the highly conserved cathelin-like prosequence and variable C-terminal sequences that correspond to the mature antibacterial peptides [18]. About 30 cathelicidin members have been identified from various mammalian species; however, only one cathelicidin, human cationic antibacterial protein of 18 kDa (hCAP18) has been found in humans, and its C-terminal mature antimicrobial peptide, called LL-37, which comprises 37 aminoacid residues (L^1^LGDFFRKSKEKIGKEFKRIVQRIKDFLRNLVPRTES^37^), has been identified [21, 22].

To date, six different human *α*-defensin molecules have been described [16, 17, 19, 20]. Human *α*-defensin-1, -2, -3, and -4 are also termed as human neutrophil peptide (HNP)-1, -2, -3, and -4, as they are mainly expressed in neutrophils. HNP-1, -2 and -3, which differ only in the first aminoacid, account for 5–7% of total neutrophil proteins, whereas HNP-4, with an aminoacid sequence distinct from other HNP sequences, comprises <1% of total defensins in neutrophils. Since no gene that encodes HNP-2 has been identified, it is regarded as a proteolytic product of HNP-1 and/or HNP-3. The other two human *α*-defensins, human defensin (HD)-5 and HD-6, are constitutively expressed in the Paneth cells within the epithelium in the small intestine. Thus, they are called enteric defensins.

Similarly, six hBDs (hBD-1 to -6) have been identified in human tissues. hBD-1 is constitutively expressed in various epithelial tissues, including urogenital and respiratory tracts [26, 27]. hBD-2, which was originally isolated from lesional scale of psoriatic skin, is mainly found in skin and respiratory as well as gastrointestinal tracts and is upregulated by various stimuli (e.g., LPS and cytokines) [28, 29]. The third *β*-defensin, hBD-3, which was also isolated from human lesional psoriatic scale, is detected in both epithelial and nonepithelial tissues, and its expression is inducible upon stimulation with bacteria and cytokines [30, 31]. hBD-4, which was initially identified by screening of human genomic sequences, is upregulated in epithelial cells by stimulation with bacteria and cytokines [32, 33]. However, the antimicrobial and other cellular activities have not yet been determined for hBD-5 and hBD-6, the newly discovered hBDs based on the human genome database [34].

In addition to their antimicrobial properties, HNPs, hBDs, and LL-37 have the potential to stimulate various host cell types to induce cytokine and chemokine production as well as to chemoattract immune and inflammatory cells [19, 35]. In this context, HNPs, hBDs, and LL-37 possess the ability to chemoattract neutrophils, monocyte, T cells, and immature dendritic cells [36–39]. Given that HNPs, hBDs, and LL-37 are multifunctional molecules as host defense peptides and act on neutrophils as chemoattractants [36, 38, 39], we hypothesized that they may have a potential to modulate the lifetime (apoptosis) of neutrophils. Here, we provided the evidence that HNP-1, hBD-3, and LL-37 can potently suppress neutrophil apoptosis, possibly via the actions on the distinct receptors; the P2Y_6_ nucleotide receptor, the CC chemokine receptor (CCR) 6, and the low-affinity formyl-peptide receptor, formyl-peptide receptor-like 1 (FPRL1)/the nucleotide receptor P2X_7_, respectively [23–25].

## 2. Modulation of Neutrophil Apoptosis by LL-37

**Figure 1:**
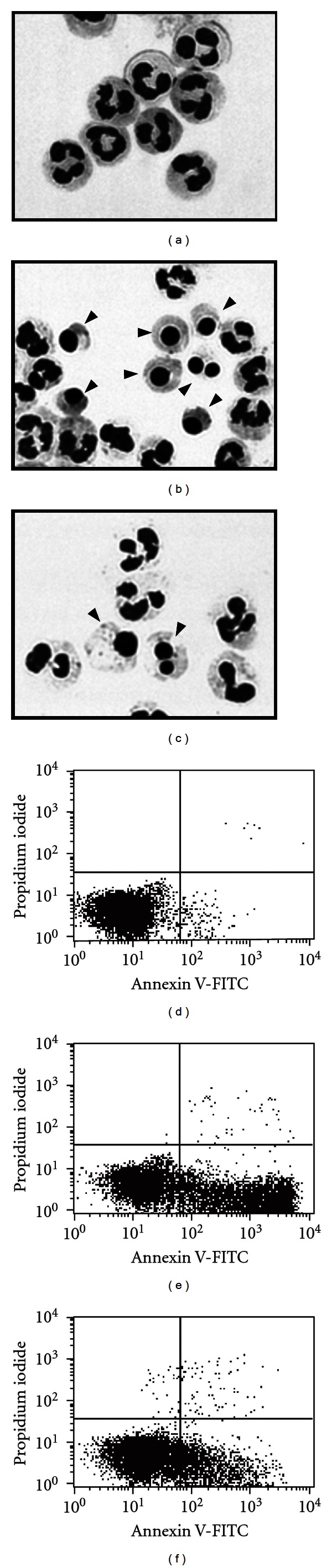
Assessment of neutrophil apoptosis by the morphological changes and FITC-annexinV/propidium iodide-staining. Neutrophils (10^6^ cells/mL) were incubated for 18 h at 37°C in RPMI1640-10%  FBS in the absence ((b) and (e)) or presence of LL-37 (1 *μ*g/mL; (c) and (f)). Neutrophils were also incubated for 18 h at 4°C in the absence of LL-37 ((a) and (d)). After incubation, neutrophils were stained with May-Grünwald-Giemsa and FITC-annexinV/propidium iodide [23–25], and neutrophil apoptosis was assessed by the morphological changes (a–c) and flow cytometry (d–f), respectively. Apoptotic neutrophils exhibit characteristic features of chromatin condensation, formation of rounded nuclear profiles, cell shrinking, and presence of cytoplasmic vacuolization (as indicated by arrowheads), and were defined as annexin V-positive but propidium iodide-negative cells. Representative data are shown.

**Figure 2:**
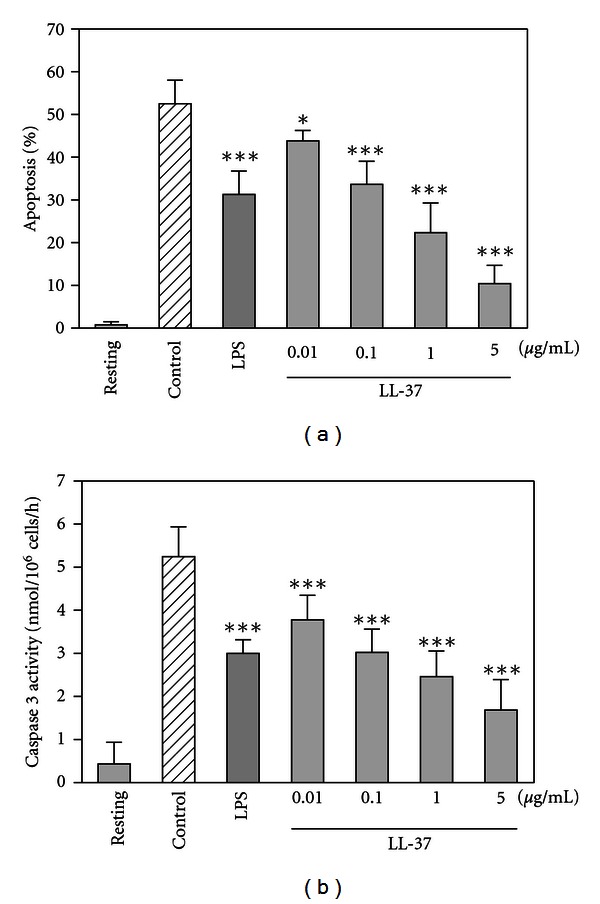
Effects of LL-37 on neutrophil apoptosis and caspase 3 activity. Neutrophils (10^6^ cells/mL) were incubated for 18 h at 37°C in RPMI1640-10% FBS in the absence (Control) or presence of LL-37 (0.01 ~ 5 *μ*g/mL) or LPS (10 ng/mL). Neutrophils were also incubated for 18 h at 4°C in the absence of LL-37 or LPS (Resting). After incubation, neutrophils were cytocentrifuged and stained with May-Grünwald-Giemsa, and a minimum of 300 neutrophils/slide was examined by light microscopy on duplicate cytospins. Apoptotic neutrophils were identified based on the morphological changes (such as chromatin condensation, rounded nuclear profiles, cell shrinking, membrane blebbing, and cytoplasmic vacuolization), quantitated and expressed as a percentage of apoptotic cells (a). Alternatively, caspase 3 activity was assayed by incubating neutrophil lysates with acetyl-Asp-Glu-Val-Asp-*p*-nitroanilide substrate in the absence or presence of acetyl-Asp-Glu-Val-Asp-al, a specific caspase 3 inhibitor at 37°C for 2 h. Caspase 3 activity is expressed as nmol of *p*-nitroanilide liberated/10^6^ cells/h (b). Data are the mean ± SD of 4 to 18 separate experiments. Values are compared between the incubation at 37°C in the absence (Control) and presence of LL-37 or LPS. All the data in this paper are analyzed for significant difference by a one-way analysis of variance (ANOVA) with multiple comparison test (Prism 4, GraphPad Software, San Diego, CA, USA). *P < 0.05; ***P < 0.001.

Before looking at the actions of LL-37, we determined the spontaneous apoptosis of neutrophils. When neutrophils were incubated alone for 18 h at 37°C, they exhibited characteristic features of apoptosis, such as chromatin condensation, formation of rounded nuclear profiles, cell shrinking, and presence of cytoplasmic vacuolization (Figure 1(b)), compared to resting cells incubated for 18 h at 4°C (Figure 1(a)). Alternatively, neutrophil apoptosis was evaluated by flow cytometry using FITC-annexin V and propidium iodide staining. Incubation of neutrophils alone for 18 h at 37°C substantially induced apoptosis defined as annexin V positive but propidium iodide negative (Figure 1(e)), compared to resting cells incubated for 18 h at 4°C (Figure 1(d)). Evaluation of neutrophil apoptosis based on the morphological changes revealed that >50% of neutrophils underwent apoptosis after incubation alone for 18 h (Figure 2(a)). LPS used as a control stimulus reduced the neutrophil apoptosis. Interestingly, spontaneous apoptosis of neutrophils was inhibited by incubation with LL-37 (Figures 1(c), 1(f), and 2(a)).

Next, we evaluated the activation of caspase 3, a key executor for apoptosis [40]. Consistent with the changes in the number of apoptotic cells, caspase 3 activity was increased after 18 h of incubation (Figure 2(b)), and the activity was reduced by LPS-stimulation. Of importance, LL-37 dose dependently suppressed the activation of caspase 3.

To clarify the mechanism for the action of LL-37, we investigated the signaling molecules by Western blot analysis. First, we looked at the effect of LL-37 on the phosphorylation of ERK, a member of mitogen-activated kinase family. LL-37 (1 *μ*g/mL) stimulation strikingly enhanced the phosphorylation of ERK-1/-2 (data not shown). Further, we evaluated the effect of LL-37 on the expression of Bcl-X_L_, an antiapoptotic protein. LL-37 (1 *μ*g/mL) markedly induced the expression of Bcl-X_L_ (data not shown).

**Figure 3:**
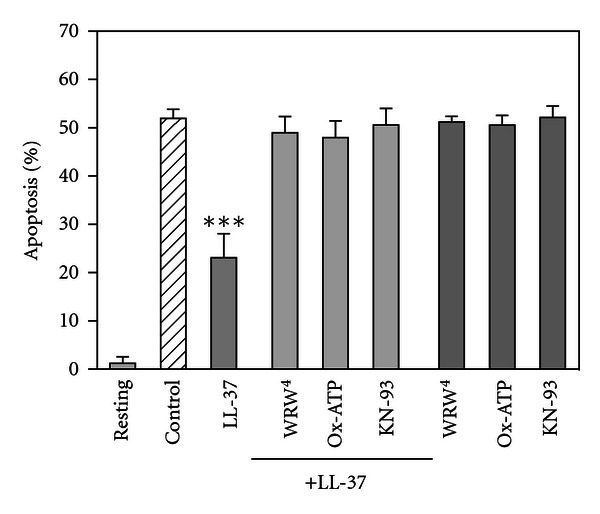
Effects of FPRL1- and P2X_7_-antagonists on the LL-37-induced suppression of neutrophil apoptosis. Neutrophils (10^6^ cells/mL) were incubated for 18 h at 37°C in RPMI1640-10% FBS in the absence (Control) or presence of LL-37 (1 *μ*g/mL), WRW^4^ (10 *μ*M), oxidized ATP (Ox-ATP, 100 *μ*M), KN-93 (5 *μ*M) or their combination (+LL-37; 10 *μ*M WRW^4^ and 1 *μ*g/mL LL-37, 100 *μ*M Ox-ATP and 1 *μ*g/mL LL-37 or 5 *μ*M KN-93 and 1 *μ*g/mL LL-37). Neutrophils were also incubated for 18 h at 4°C in the absence of LL-37, FPRL1-, or P2X_7_-antagonists (Resting). After incubation, apoptosis of neutrophils was quantitated and expressed as a percentage of apoptotic cells. Data are the mean ± SD of 4 to 12 separate experiments. Values are compared between the incubation at 37°C in the absence (Control) and presence of LL-37, WRW^4^, oxidized ATP, KN-93 or their combination. ***P < 0.001.

It has been demonstrated that LL-37 uses FPRL1 as a receptor to chemoattract neutrophils, monocytes and T cells [38]. In addition, LL-37 is reported to promote the processing and release of IL-1*β* from monocytes via the activation of P2X_7_ receptor [41]. Thus, we determined the involvement of FPRL1 and P2X_7_ in the LL-37-induced suppression of neutrophil apoptosis by using FPRL1 antagonist and P2X_7_ inhibitors. As shown in Figure 3, antagonistic agents for FPRL1 (WRW^4^, Trp-Arg-Trp-Trp-Trp-Trp-CONH_2_) [42] and P2X_7_ (oxidized ATP and KN-93) [43–45] significantly reversed the LL-37-induced suppression of neutrophil apoptosis. Similarly, FPRL1 antagonist (WRW^4^) and P2X_7_ inhibitors (oxidized ATP and KN-93) obviously attenuated the LL-37-induced inhibition of caspase 3 activity (data not shown). These observations apparently suggest that FPRL1 and P2X_7_ are involved in the LL-37-induced suppression of neutrophil apoptosis.

**Figure 4:**
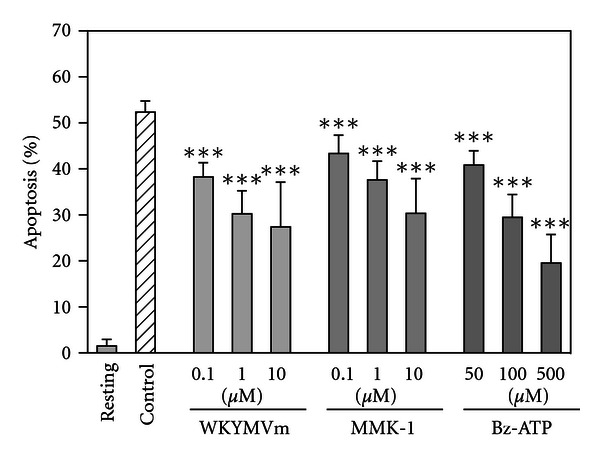
Effects of FPRL1- and P2X_7_-agonists on neutrophil apoptosis. Neutrophils (10^6^ cells/mL) were incubated for 18 h at 37°C in RPMI1640-10%  FBS in the absence (Control) or presence of WKYMVm (0.1 ~ 10 *μ*M), MMK-1 (0.1 ~ 10 *μ*M) or Bz-ATP (50 ~ 500 *μ*M). Neutrophils were also incubated for 18 h at 4°C in the absence of FPRL1- or P2X_7_-agonists (Resting). After incubation, apoptosis of neutrophils was quantitated and expressed as a percentage of apoptotic cells. Data are the mean ± SD of 4 to 15 separate experiments. Values are compared between the incubation at 37°C in the absence (Control) and presence of WKYMVm, MMK-1, or Bz-ATP. *P < 0.05; ***P < 0.001.

Next, to further determine the involvement of FPRL1 and P2X_7_ in the suppression of neutrophil apoptosis, neutrophils were directly incubated with the FPRL1 and P2X_7_ agonists. As shown in Figure 4, agonistic agents for FPRL1 (WKYMVm, Trp-Lys-Tyr-Met-Val-D-Met-CONH_2_ and MMK-1, LESIFRSLLFRVM) [46], and P2X_7_ (Bz-ATP, benzoylbenzoyl-ATP) [43, 44] dose dependently suppressed neutrophil apoptosis. Notably, the combinations of FPRL1- and P2X_7_-agonists cooperatively suppressed neutrophil apoptosis as well as the activation of caspase 3 (data not shown). These observations most likely indicate that the activation of FPRL1 and P2X_7_ in concert acts to induce the suppression of neutrophil apoptosis. 

## 3. Modulation of Neutrophil Apoptosis by hBDs

**Figure 5:**
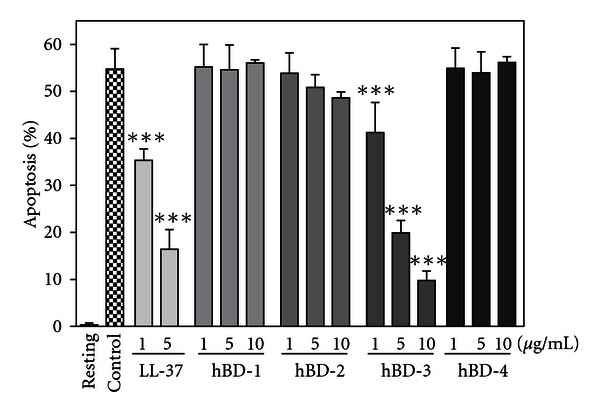
Effects of hBDs on neutrophil apoptosis. Neutrophils (10^6^ cells/mL) were incubated at 37°C for 18 h in RPMI1640-10%  FBS in the absence (Control) or presence of hBD-1, -2, -3, and -4 (1, 5 and 10 *μ*g/mL), or LL-37 (1 and 5 *μ*g/mL). Neutrophils were also incubated at 4°C for 18 h in the absence of hBDs or LL-37 (Resting). After incubation, neutrophil apoptosis was quantitated and expressed as a percentage of apoptotic cells. Data are the mean ± SD of 3 to 25 separate experiments. Values are compared between the incubation at 37°C in the absence (Control) and presence of hBDs or LL-37. ***P < 0.001.

Next, we determined the effect of HBDs on neutrophil apoptosis. Interestingly, hBD-3 dose dependently inhibited the neutrophil apoptosis (Figure 5). Of note, neither hBD-1, hBD-2 nor hBD-4 significantly influenced neutrophil apoptosis at the concentrations examined. Similarly, hBD-3 but not hBD-1, hBD-2 and hBD-4 significantly suppressed the activation of caspase 3 (data not shown).

**Figure 6:**
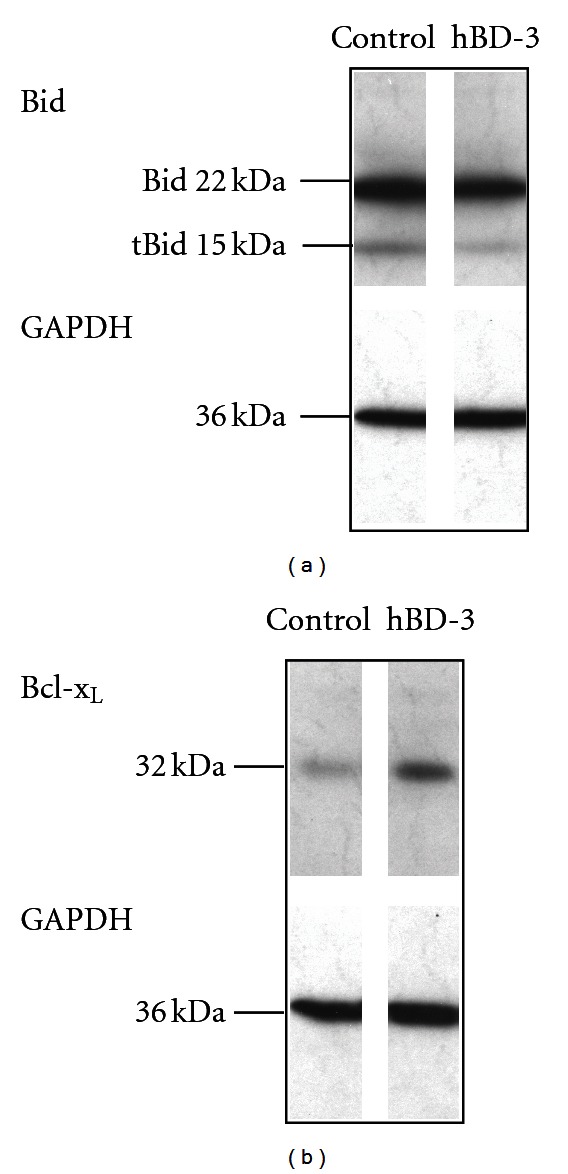
Effects of hBD-3 on the expression of truncated Bid and Bcl-x_L_. Neutrophils (10^6^ cells/mL) were incubated at 37°C for 4 h in RPMI1640-10%  FBS in the absence (Control) or presence of hBD-3 (5 *μ*g/mL). Expression of truncated Bid (tBid) and Bcl-x_L_ was detected by probing with goat anti-human/mouse Bid polyclonal Ab (a) or mouse anti-Bcl-x_L_ mAb (b), and HRP-conjugated rabbit anti-goat IgG or HRP-conjugated goat anti-mouse IgG/IgM, respectively. To confirm that equal amounts of proteins were analyzed in each sample, the blots were stripped, and GAPDH was detected by reprobing with mouse anti-GAPDH mAb and HRP-conjugated goat anti-mouse IgG/IgM. Anti-human/mouse Bid polyclonal Ab can recognize 17-kDa truncated Bid (tBid) as well as 22-kDa Bid (upper half of panel (a)). Data are from 1 of 4 separate experiments.

Further, we evaluated the expression of apoptosis-associated proteins, truncated Bid (a proapoptotic protein) and Bcl-x_L_ (an antiapoptotic protein). Of note, consistent with its suppressive action on neutrophil apoptosis, hBD-3 downregulated truncated Bid, whereas it upregulated Bcl-x_L_ (Figure 6).

**Figure 7:**
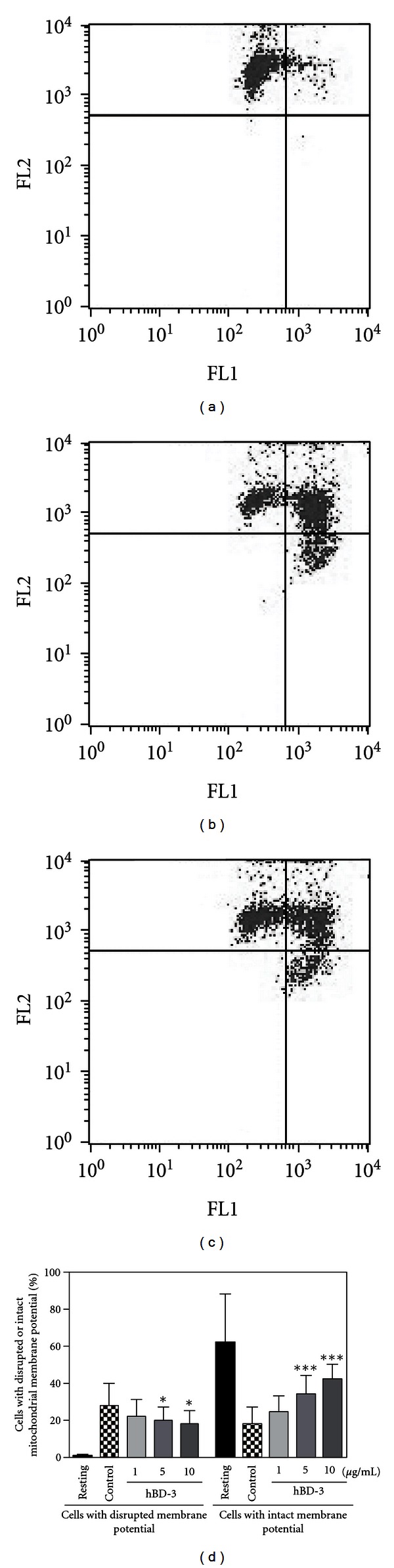
Effects of hBD-3 on the mitochondrial membrane potential change. Neutrophils (10^6^ cells/mL) were incubated at 37°C for 18 h in RPMI1640-10%  FBS in the absence ((b); Control) or presence of 1 ~ 10 *μ*g/mL hBD-3 ((c), 5 *μ*g/mL). Neutrophils were also incubated at 4°C for 18 h in the absence of hBDs ((a); Resting). After incubation, cells were harvested and stained with a mitochondrial membrane potential-dependent lipophilic dye, JC-1. The stained cells were analyzed for the intensities of green fluorescence (FL1) and red fluorescence (FL2) by flow cytometry. Results were expressed as a percentage of cells with disrupted (enhanced green but diminished red fluorescence) or intact (enhanced red but diminished green fluorescence) mitochondrial membrane potential (d). Data are the mean ± SD of 9 to 12 separate experiments. Values are compared between the incubation at 37°C in the absence (Control) and presence of 1 ~ 10 *μ*g/mL hBD-3. *P < 0.05; ***P < 0.001.

The dissipation of mitochondrial electrochemical potential gradient is known as an early event of apoptosis [47]. Thus, we investigated the effect of hBD-3 on the mitochondrial membrane potential change in apoptotic neutrophils. Assessment of the mitochondrial membrane potential change with a cationic lipophilic dye JC-1 [47] revealed that the percentage of cells with intact mitochondrial membrane potential (enhanced red but diminished green fluorescent cells) decreased, whereas that with disrupted mitochondrial membrane potential (enhanced green but diminished red fluorescent cells) increased after incubation at 37°C for 18 h (Figure 7). Importantly, hBD-3 suppressed the mitochondrial membrane potential change; hBD-3 (1 ~ 10 *μ*g/mL) dose dependently decreased neutrophil number with disrupted mitochondrial membrane potential, but increased that with intact mitochondrial membrane potential even after incubation at 37°C for 18 h.

**Figure 8:**
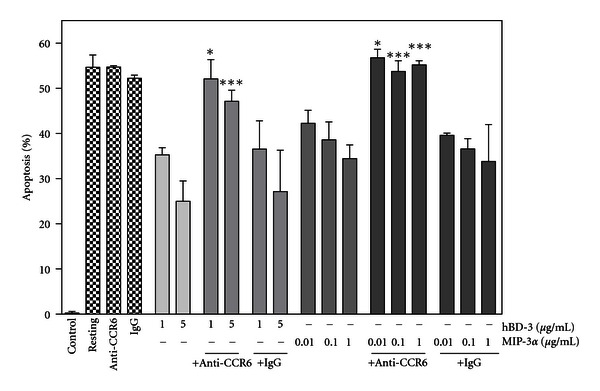
Effects of a neutralizing anti-CCR6 mAb on the hBD-3- and MIP-3*α*-induced suppression of neutrophil apoptosis. Neutrophils (10^6^ cells/mL) were incubated at 37°C for 18 h in RPMI1640-10% FBS in the absence (Control) or presence of hBD-3 (1 and 5 *μ*g/mL), MIP-3*α* (0.01, 0.1, and 1 *μ*g/mL), anti-CCR6 mAb (50 *μ*g/mL), control IgG (50 *μ*g/mL), or their combination (+ Anti-CCR6; 1 ~ 5 *μ*g/mL hBD-3 and anti-CCR6 mAb, or 0.01 ~ 1 *μ*g/mL MIP-3*α* and anti-CCR6 mAb; + IgG; 1 ~ 5 *μ*g/mL hBD-3 and control IgG, or 0.01 ~ 1 *μ*g/mL MIP-3*α* and control IgG). Neutrophils were also incubated at 4°C for 18 h in the absence of hBD-3, MIP-3*α*, anti-CCR6 mAb, or control IgG (Resting). After incubation, neutrophil apoptosis was quantitated and expressed as a percentage of apoptotic cells. Data are the mean ± SD of 3 to 6 separate experiments. Values are compared between the incubation at 37°C in the presence of hBD-3 (1 and 5 *μ*g/mL) or MIP-3*α* (0.01, 0.1 and 1 *μ*g/mL) without and with anti-CCR6 mAb or control IgG. *P < 0.05; ***P < 0.001.

Although CCR6 is known as a specific receptor for CCL20/MIP-3*α* [48], hBD-3 also utilizes CCR6 to stimulate chemotaxis for monocytes and CCR6-transfected human embryonic kidney (HEK) 293 cells [49]. Thus, we determined the participation of CCR6 in the hBD-3-induced suppression of neutrophil apoptosis. When neutrophils were incubated with hBD-3 in the presence of anti-CCR6 mAb or control IgG, anti-CCR6 mAb but not control IgG significantly reversed the hBD-3-induced suppression of neutrophil apoptosis (Figure 8). To further clarify the involvement of CCR6 in the suppression of neutrophil apoptosis, neutrophils were incubated with MIP-3*α*, a specific ligand for CCR6 [48]. MIP-3*α* (0.01 ~ 1 *μ*g/mL) dose dependently suppressed neutrophil apoptosis, and MIP-3*α*-induced suppression of neutrophil apoptosis was obviously attenuated by anti-CCR6 mAb but not control IgG. These observations suggest that CCR6 is involved in not only the MIP-3*α*-induced but also the hBD-3-induced suppression of neutrophil apoptosis.

## 4. Modulation of Neutrophil Apoptosis by *α*-Defensins

**Figure 9:**
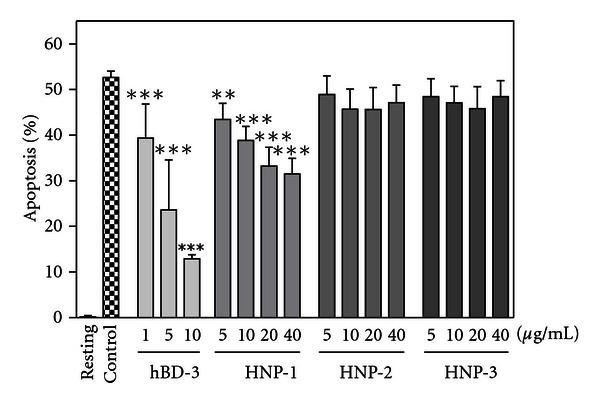
Effects of HNPs on neutrophil apoptosis. Neutrophils (10^6^ cells/mL) were incubated at 37°C for 18 h in RPMI1640-10%  FBS in the absence (Control) or presence of HNP-1, -2, and -3 (5, 10, 20, and 40 *μ*g/mL), or hBD-3 (1, 5 and 10 *μ*g/mL). Neutrophils were also incubated at 4°C for 18 h in the absence of HNPs or hBD-3 (Resting). After incubation, neutrophil apoptosis was quantitated and expressed as a percentage of apoptotic cells. Data are the mean ± SD of 3 to 12 separate experiments. Values are compared between the incubation at 37°C in the absence (Control) and presence of HNPs or hBD-3. **P < 0.01, ***P < 0.001.

Further, we determined the effect of *α*-defensins on neutrophil apoptosis. Interestingly, HNP-1 (5 ~ 40 *μ*g/mL) dose dependently suppressed neutrophil apoptosis (Figure 9). However, HNP-2, HNP-3 and HD-5 (data not shown) did not significantly influence the neutrophil apoptosis at the concentrations examined. Similarly, HNP-1 but not HNP-2, HNP-3, and HD-5 significantly suppressed the activation of caspase-3 (data not shown). Moreover, consistent with its suppressive action on neutrophil apoptosis, HNP-1 downregulated truncated Bid, whereas it upregulated Bcl-x_L_ (data not shown). Importantly, we confirmed that HNP-1 suppressed the mitochondrial membrane potential change; HNP-1 (40 *μ*g/mL) increased neutrophil number with intact mitochondrial membrane potential, but decreased that with disrupted mitochondrial membrane potential even after incubation at 37°C for 18 h (data not shown).

**Figure 10:**
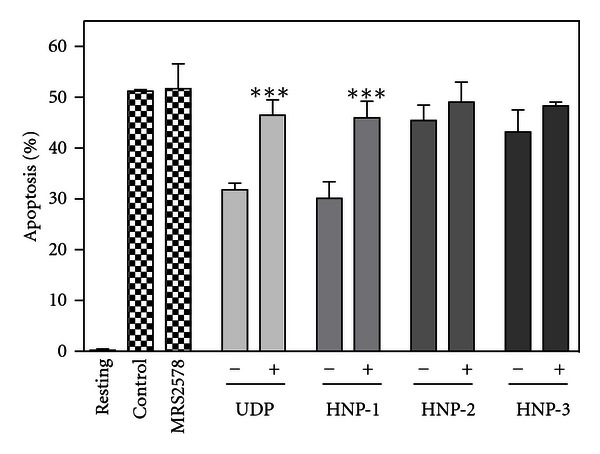
Effect of a P2Y_6_ antagonist on the HNP-1-induced suppression of neutrophil apoptosis. Neutrophils (10^6^ cells/mL) were incubated for 18 h at 37°C in RPMI1640-10%  FBS in the absence (Control) or presence of HNP-1, -2, and -3 (20 *μ*g/mL each), UDP (300 *μ*M), MRS2578 (1 *μ*M), or their combination (+; 20 *μ*g/mL HNP-1, -2, or -3 and 1 *μ*M MRS2578, or 300 *μ*M UDP, and 1 *μ*M MRS2578). Neutrophils were also incubated alone for 18 h at 4°C in the absence of HNPs, UDP or MRS2578 (Resting). After incubation, apoptosis of neutrophils was quantitated and expressed as a percentage of apoptotic cells. Data are the mean ± SD of 3 to 5 separate experiments. Values are compared between the incubation at 37°C with HNPs or UDP in the absence (−) and presence (+) of MRS2578 (d). **P < 0.01; ***P < 0.001.

Although P2Y_6_ is known as a purinergic receptor for UDP [50], it has been reported that HNPs utilizes P2Y_6_ signaling to stimulate human lung epithelial cells to produce IL-8 [51]. Thus, we determined the involvement of P2Y_6_ in the HNP-1-induced suppression of neutrophil apoptosis. To determine the involvement of P2Y_6_ in the suppression of neutrophil apoptosis, neutrophils were directly incubated with a P2Y_6_ agonist UDP, and its effect on neutrophil apoptosis was evaluated. UDP (30 ~ 3000 *μ*M) dose dependently suppressed neutrophil apoptosis (data not shown). Furthermore, we evaluated the effects of a P2Y_6_ antagonist MRS2578 [52]. Of importance, MRS2578 significantly reversed the HNP-1-induced as well as UDP-induced suppression of neutrophil apoptosis (Figure 10). These observations suggest that P2Y_6_ is involved in not only the UDP-induced but also the HNP-1-induced suppression of neutrophil apoptosis.

**Figure 11:**
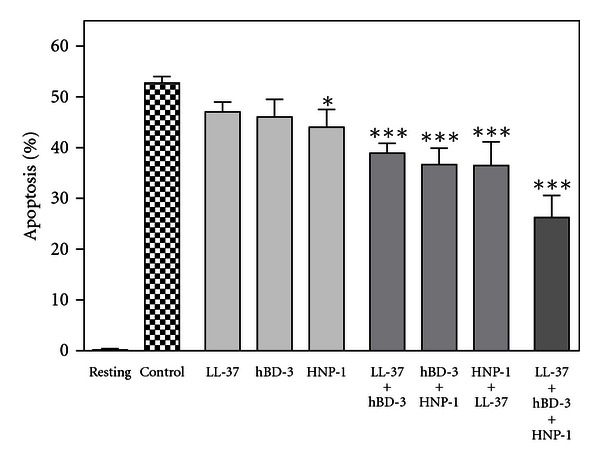
Effects of LL-37, hBD-3 and HNP-1 on neutrophil apoptosis. Neutrophils (10^6^ cells/mL) were incubated for 18 h at 37°C in RPMI1640-10%  FBS in the absence (Control) or presence of LL-37 (0.1 *μ*g/mL), hBD-3 (1 *μ*g/mL), HNP-1 (10 *μ*g/mL), or their combination (0.1 *μ*g/mL LL-37 and 1 *μ*g/mL hBD-3, 1 *μ*g/mL hBD-3 and 10 *μ*g/mL HNP-1, 10 *μ*g/mL HNP-1 and 0.1 *μ*g/mL LL-37, or 0.1 *μ*g/mL LL-37, 1 *μ*g/mL hBD-3 and 10 *μ*g/mL HNP-1). Neutrophils were also incubated alone for 18 h at 4°C in the absence of LPS (Resting). After incubation, neutrophil apoptosis was quantitated and expressed as a percentage of apoptotic cells. Data are the mean ± SD of 4 to 5 separate experiments. Values are compared between the absence (Control) and presence of LL-37, hBD-3, HNP-1, or their combination. *P < 0.05, ***P < 0.001.

We demonstrated that the human antimicrobial peptides, LL-37, hBD-3, and HNP-1 can suppress neutrophil apoptosis. Thus, we determined whether LL-37, hBD-3, and HNP-1 could cooperatively suppress the neutrophil apoptosis by incubating neutrophils with the combinations of LL-37, hBD-3, and HNP-1. As shown in Figure 11, the low concentrations of LL-37 (0.1 *μ*g/mL), hBD-3 (1 *μ*g/mL), and HNP-1 (10 *μ*g/mL) slightly reduced the neutrophil apoptosis. Of note, the combinations of 2 peptides cooperatively decreased apoptosis, and the combination of 3 peptides further reduced apoptosis. These observations clearly indicate that LL-37, hBD-3, and HNP-1 can act in concert on neutrophils to suppress the apoptosis.

## 5. Perspectives

Host defense peptides (*α*- and *β*-defensins; cathelicidins), as the effectors in the innate host defense system, exhibit antimicrobial activities against a broad spectrum of microbes, including both Gram-positive and Gram-negative bacteria, fungi, and viruses [15]. In addition to their antimicrobial properties, HNPs, hBDs, and LL-37 have the potential to stimulate various host cell types to induce cytokine and chemokine production [19, 35]. Furthermore, HNPs, hBDs, and LL-37 possess the ability to chemoattract neutrophils, monocyte, T cells, and immature dendritic cells [36–39].

**Figure 12:**
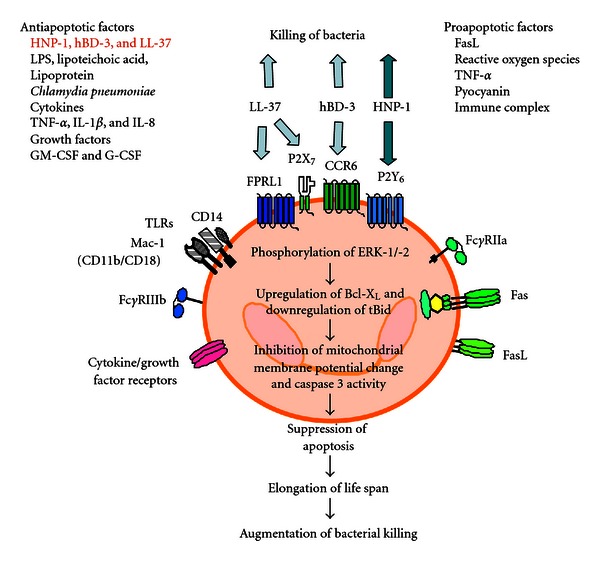
Summary of HNP-1-, hBD-3-, and LL-37-induced suppression of neutrophil apoptosis. The lifetime of neutrophils, terminally differentiated blood cells, is relatively short, and is regulated by various pathogen- and host-derived antiapoptotic and proapoptotic substances [1–14]. Antimicrobial host defense peptides, HNP-1, hBD-3, and LL-37 cannot only destroy bacteria but also potently suppress neutrophil apoptosis, accompanied with the phosphorylation of ERK-1/-2, the downregulation of tBid (an proapoptotic protein) and upregulation of Bcl-x_L_ (an antiapoptotic protein), and the inhibition of mitochondrial membrane potential change and caspase 3 activity, possibly via the actions on the distinct receptors, the P2Y_6_ nucleotide receptor, the chemokine receptor CCR6, and the low-affinity formyl-peptide receptor FPRL1/the nucleotide receptor P2X_7_, respectively [23–25]. Suppression of neutrophil apoptosis results in the prolongation of their lifespan and may be advantageous for the host defense against bacterial invasion.

In this paper, we demonstrated that HNP-1, hBD-3, and LL-37 can potently suppress neutrophil apoptosis among the peptides examined. During the process of apoptosis, truncated Bid (a proapoptotic protein) is cleaved from Bid and translocates to mitochondria to perturb the mitochondrial functions, thereby disrupting mitochondrial membrane potential and promoting cytochrome c release, which results in the activation of effector caspases and finally induces apoptosis [40, 53]. In contrast, Bcl-x_L_ acts as an antiapoptotic protein to preserve mitochondrial integrity, thereby suppressing the activation of caspase cascade and apoptosis [40, 54]. Consistent with their antiapoptotic actions, HNP-1, hBD-3, and LL-37 downregulated truncated Bid and upregulated Bcl-x_L_. Furthermore, they inhibited the dissipation of mitochondrial membrane potential and activation of caspase 3, one of the death proteases functioning as the central executioners of apoptosis [40]. It has been shown that the activation of ERK, a member of mitogen-activated kinase family, generates the survival signals via the upregulation of antiapoptotic proteins of Bcl-2 family (such as Bcl-X_L_) to prolong the life span of cells [3, 55]. Since we confirmed that HNP-1 and hBD3 as well as LL-37 induced the phosphorylation of ERK-1/-2 in neutrophils, it is feasible to assume that the stimulation of neutrophils with HNP-1, hBD-3, and LL-37 induces the phosphorylation of ERK-1/-2 and the subsequent upregulation of antiapoptotic protein Bcl-X_L_ and downregulation of proapoptotic protein truncated Bid, which inhibits the mitochondrial membrane potential change and caspase 3 activity, thereby suppressing neutrophil apoptosis (Figure 12).

Cationic antimicrobial peptides (such as defensins and cathelicidins) kill the invaded microorganisms by perturbing their membranes; the action of those peptides is not receptor-mediated but involves a less specific interaction with microbial membrane components, since the peptides target cell surface anionic lipids such as phosphatidyl glycerol and cardiolipin that are abundant in microorganisms [56]. In contrast, the mammalian cell membrane is mainly composed of electrically neutral phospholipids such as phosphatidylcholine and sphingomyelin, for which the affinity of antimicrobial peptides is generally low [56]. Recently, antimicrobial peptides have been shown to act on the cell surface receptors to modulate various host cell functions (i.e., proinflammatory mediator production; immune and inflammatory cell activation). For instance, HNPs induce IL-8 production from A549 lung epithelial cells via the action on P2Y_6_ nucleotide receptor [51], and hBD-3 utilizes a chemokine receptor CCR6 to chemoattract monocytes and CCR6-transfected HEK 293 cells [49]. Moreover, LL-37 functions as a chemoattractant for neutrophils, monocytes, and T cells via the interactions with FPRL1, a low-affinity formyl-peptide receptor [38] and promotes the processing and release of IL-1*β* from monocytes via the activation of P2X_7_ nucleotide receptor [41]. Using antagonistic and agonistic agents and a neutralizing antibody, we demonstrated that P2Y_6_, CCR6, and FPRL1/P2X_7_ are likely involved in the HNP-1-, hBD-3, and LL-37-induced suppression of neutrophil apoptosis, respectively. To date, it is proposed that LL-37 and hBD-3 can directly bind to and activate FPRL1 and CCR6 [24, 46]. However, the exact mechanisms by which LL-37 and HNPs signal through the P2Y_7_ and P2Y_6_ receptors remain to be elucidated [41, 51]. The current study demonstrated that among *α*-defensins, HNP-1 but not HNP-2, HNP-3 and HD-5 suppresses the apoptosis of neutrophils via the P2Y_6_ signaling. Of interest, aminoacid sequences of HNP-1, -2, and -3 differ only in the *N*-terminal end, whereas HD-5 is distinct from other HNPs. Thus, it can be postulated that the difference in the *N*-terminal sequences of HNP-1 ~ -3 can be recognized by the P2Y_6_ signaling to induce the suppression of neutrophil apoptosis. Supporting this sequence-specific actions of HNPs, it has been reported that HNP-1 most potently exhibits the chemotactic activity for monocytes among HNP-1 ~ -3 [36].

HNPs are synthesized in neutrophils, whereas hBDs are mainly produced in epithelial tissues, including respiratory and urogenital tracts and skin [16, 17, 19, 20]. Furthermore, LL-37 is expressed in keratinocytes and lung epithelial cells as well as neutrophils [19, 57]. Interestingly, the expression of these antimicrobial peptides is locally induced at the sites of inflammation and infection within epithelial cells, and invading neutrophils represent an additional source for the peptides [57, 58]. In this context, it has been reported that the concentrations of HNPs, hBDs, and LL-37 are increased up to 40 *μ*g/mL in bronchoalveolar and nasal fluids from patients with inflammation and infection [58–60]. Importantly, we demonstrated that LL-37, hBD-3, and HNP-1 can suppress neutrophil apoptosis *in vitro* at the concentrations (0.01 ~ 40 *μ*g/mL) comparable to those at the sites of inflammation and infection, and that LL-37, hBD-3, and HNP-1 can cooperatively suppress neutrophil apoptosis *in vitro*. Thus, it can be speculated that antimicrobial peptides LL-37, hBD-3, and HNP-1, in concert modulate neutrophil apoptosis *in vivo* in the local milieu at the sites of inflammation or infection by utilizing different receptors (e.g., FPRL1, P2X_7_, CCR6, and P2Y_6_). Furthermore, HNPs, and LL-37 are expected to exert their actions on neutrophils in a paracrine/autocrine fashion, as they are stored in the azurophil and specific granules of neutrophils, respectively, and extracellularly released from activated neutrophils [61, 62]. In this context, it should be noted that antiapoptotic genes are upregulated, and proapoptotic genes are downregulated *in vivo* in neutrophils that challenged with inflammatory/immunomodulatory molecules (including LL-37, hBDs, and HNPs), and transmigrated to the inflammatory skin lesions [63].

Clearance of neutrophils from inflamed tissues is critical for the resolution of inflammation. Clinical studies have indicated that spontaneous neutrophil apoptosis is inhibited in patients with severe inflammation (e.g., sepsis, SIRS and ARDS) by the actions of various bacterial products, cytokines and chemokines, detected in these disorders [3, 5–8]. Activated neutrophils with prolonged survival cause the amplification of inflammation and tissue injury via the uncontrolled release of cytotoxic metabolites and proinflammatory substances [9, 10]. From this point of view, antimicrobial peptides (LL-37, hBD-3, and HNP-1) may exert a harmful effect during inflammation by suppressing apoptosis and prolonging the lifespan (survival) of neutrophils, which could lead to the augmented inflammatory reactions. In contrast, physiological process of neutrophil apoptosis can be subverted by bacterial pathogens during infections [64]. Inappropriate or premature apoptosis of neutrophils could deplete cell numbers and functions, impairing host defense and favoring bacterial persistence in infections. In this context, it has been reported that neutrophil apoptosis is accelerated and neutrophil-mediated host defense is impaired *in vivo* during infection with *Pseudomonas aeruginosa* by the action of pyocyanin, a predominant phenazine exotoxin [14]. Considering their antiapoptotic action, antimicrobial peptides (LL-37, hBD-3, and HNP-1) can exert an advantageous effect on host defense against bacterial infections by prolonging the lifespan of neutrophils, major phagocytes engaged in the killing of invading bacteria.

HNPs, hBDs, and LL-37 are originally identified as antimicrobial peptides, which participate in the innate immune system, capable of protecting host from invasive microbial infections [16, 17], and now regarded as multifunctional molecules that link the innate immune response to the adaptive immune system by exerting various immunomodulatory actions, such as cytokine and chemokine production, and immune and inflammatory cell migration [19, 35]. In this paper, we have demonstrated an additional function of HNPs, hBDs, and LL-37 to prolong the lifespan of neutrophils via the actions on the distinct receptors. This finding provides a novel insight into the role of antimicrobial peptides in the regulation of neutrophil lifespan (apoptosis) as well as the innate and adaptive host defense systems.
